# A randomized controlled trial on effects of different hemostatic sponges in posterior spinal fusion surgeries

**DOI:** 10.1186/s12893-016-0197-3

**Published:** 2016-12-12

**Authors:** Derong Xu, Zhinan Ren, Xin Chen, Qianyu Zhuang, Lin Sheng, Shugang Li

**Affiliations:** Department of Orthorpaedic Surgery, Peking Union Medical College Hospital, No.1 Shuai Fu Yuan, Wang Fu Jing Street, Beijing, 100730 People’s Republic of China

**Keywords:** Lumbar fusion surgery, Hemostatic collagen sponge, Gelatin sponge, Blood loss

## Abstract

**Background:**

Spinal fusion surgery is associated with significant blood loss, which may result in potential clinical complications, it is necessary to take safe and effective measures to reduce blood loss in surgery. We perform this study to assess the impact of three different hemostatic materials on perioperative blood loss.

**Methods:**

We performed a Randomized Controlled Trial research and recruited patients with lumbar disease into the study between November 2013 and March 2015. All the participants were randomly assigned to 3 groups using a simple equal probability randomization scheme: Group A (Stypro hemostatic sponge), Group B (Collagen hemostatic sponge) and Group C (gelatin sponge). We compared postoperative blood loss between these 3 groups.

**Results:**

In our study, drainage volume in the first 24 h of patients in Group A and B is significantly smaller, as well as total postoperative volumes of drainage (*p* < 0.05) during their hospital stay. The drainage volumes in the second 24 h were similar in the 3 groups. We also found that the average drainage Hematocrit (HCT) reduced over time, the average HCT of drainage is 18.04% and 11.72% on the first day and on the second day respectively.

**Conclusions:**

Hemostatic collagen sponge demonstrated better hemostasis effects than gelatin sponge with lower volume of postoperative drainage volume and blood loss in posterior spinal fusion surgery.

**Trial registration:**

The trial registration number (TRN) of the study is ISRCTN29254316 and date of registration is 25/10/2016. Our trial was registered retrospectively.

## Background

Spinal fusion surgeries may result in significant blood loss, which is often associated with cardiovascular complications and high rates of allogeneic blood transfusion [1]. Therefore, it is imperative to take safe and effective measures to reduce blood loss and rates of transfusions after spinal fusion surgeries. In recent years, the use of hemostatic materials, such as gelatin sponge and other collagen sponges, has led to significant reductions in both intraoperative blood loss and postoperative transfusions [2]. However, there are many differences between gelatin sponges, which have been in use for several decades, and collagen sponges which have been featured with new properties. The objective of our study was to assess the impact of three different hemostatic materials on operative blood loss.

## Methods

Our study is a randomized controlled trial. The Stypro hemostatic sponge used in this study mainly consisted of medicinal protein collected from pigs. The Collagen hemostatic sponge is also a product derived from animal original type I collagen from cows. Both of them possess such biological properties that can activate the intrinsic coagulation pathway. The gelatin sponges, which have no bioactivity, control bleeding mainly by volume expansion and mechanical compression.

This RCT study was carried out at PUMC Hospital. We recruited patients with lumbar diseases into the study from November, 2013 to March, 2015. The inclusion criteria were lumbar stenosis, disc disease, and instability (e.g. grade I-II spondylolisthesis, spondylolisthesis /spondylolysis) which were indicated for spinal surgeries. The exclusion criteria were as follows: 1. severe medical comorbidities such as osteoporosis, anemia and cardiovascular disease. 2. Involvement of more than three surgical levels 3. Patients had abnormal prothrombin time (PT), partial thromboplastin time (PTT) and International Normalized Ratio (INR) 4. Patients were taking anti-platelet aggregates such as Aspirin or other anticoagulants.

**Fig. 1:**
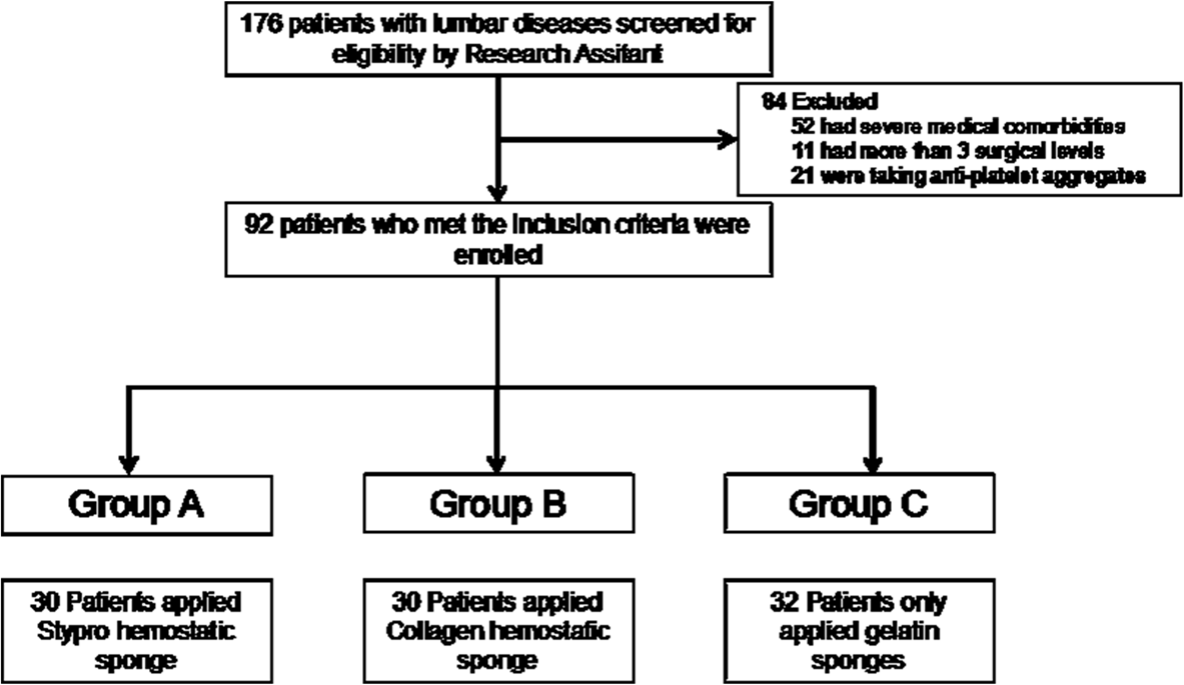
CONSORT flow diagram

All the participants were randomly assigned to 3 groups using a simple equal probability randomization scheme: Group A (Stypro hemostatic sponge group), Group B (Collagen hemostatic sponge group) and Group C (gelatin sponge group). The participants were presented in a flow diagram in Fig. 1.

All the patients were performed posterior lumbar decompression, internal fixation and bone graft fusion surgery by the same surgeon. In addition, patients underwent discectomy if diagnosed with disc herniation and reduction if accompanied with spondyloliosthesis.

After the decompression, we controlled the bleeding meticulously with use of bipolar electrocautery, after which we placed different sponges on the surface of spinal dura mater respectively. We applied hemostatic sponges with two different brands in group A (Stypro hemostatic sponge) and group B (Collagen hemostatic sponge), as for group C, we used gelatin sponges. According to the size of exposed spinal dura, we cut the hemostatic materials into proper corner to ensure that the entire dura would be covered.

Intraoperative estimated blood loss (EBL) were calculated on the basis of surgical sponges soaked and volume in suction canisters, subtracting irrigation fluid added to the surgical field [3]. Deep drainage was placed below the fascia in all patients at the end of operations. We recorded the amount of postoperative drainage in the first 24 h, the second 24 h and the total drainage volume. In addition, complete blood count (CBC) was examined and analyzed for every drainage sample to obtain data of HCT and Hemoglobin (HGB), which were used to calculate the blood contained in drainage.

The drainage was routinely removed when the drain output per 24 h was <50 ml. Clinical data, including age, height, weight, body mass index (BMI), operative durations, surgical levels, intraoperative blood loss, related complications, and length of hospital stay were compared between the three groups. Besides, three parameters were compared between 3 groups: (1) volume of drainage in the first and second 24 h, and patient’s total drain output (2) HCT of drainage in the first and second 24 h (3) drainage blood at different time points.

This prospective study was approved by the ethical committee at Peking Union Medical College Hospital, and all participants provided written informed consents for the study and surgery.

The difference in demographic and perioperative data between 3 groups was analyzed using the One-Way ANOVA. In all analyses, the level of statistical significance was set at *P* < 0.05. All data analyses were performed with the SPSS 19.0 software package.

## Results

In this study, 92 patients who met the inclusion criteria were enrolled. Among these patients, 30 patients were assigned to group A, 30 patients were assigned to group B and 32 patients were assigned to group C. No significant difference in age, height, weight, BMI, surgical level, intraoperative blood loss, operative durations, the mean length of hospital stay and coagulation indices were identified between the 3 groups, as presented in Table 1.

**Table 1:** Demographic date

Variable	Group A	Group B	Group C
N	30	30	32
Age (year)	56.1 ± 12.9	55.6 ± 12.3	56.7 ± 13.4
Sex			
Males	12	13	13
Females	18	17	19
BMI (kg/cm2)	24.83 ± 3.6	24.93 ± 3.3	24.81 ± 3.4
Preoperative HGB (g/L)	123.2 ± 10.5	125.1 ± 9	126.4 ± 8.4
Surgical level	2.31 ± 0.09	2.76 ± 0.12	2.40 ± 0.10
Operative time (min)	118.0 ± 24	150.6 ± 47	135.6 ± 39
Blood loss (ml)	202.5 ± 25	292.7 ± 39	259.8 ± 37
Mean duration of hospital stay (days)	6.82 ± 1.2	6.89 ± 1.3	7.3 ± 1.3

**Fig. 2:**
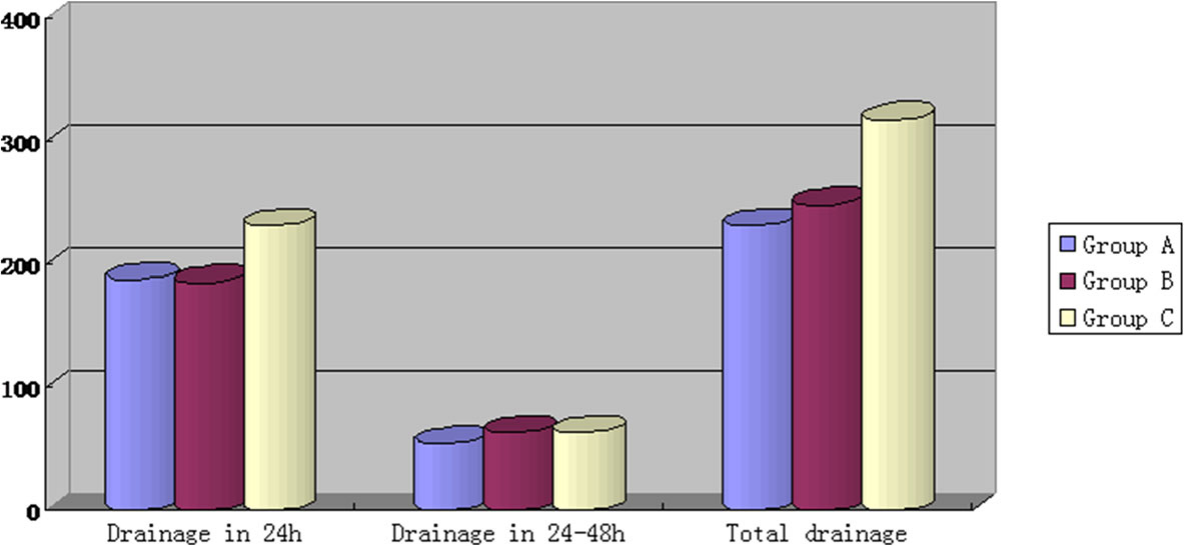
Comparison of postoperative drainage

The detailed information of drainage at various time points is presented in Table 2 and Fig. 2. Compared with patients in Group C, patients in Group A and B exhibited significantly smaller drainage volume in the first 24 h, as well as total postoperative volumes of drainage (*p* < 0.05) during their hospital stay. The drainage volumes in the second 24 h were similar in the 3 groups and exhibited no significant differences.

**Table 2:** The information of postoperative drainage

Variable	Group A	Group B	Group C
Volume of Drainage in 24 h (ml)*	187.9 ± 82	185.2 ± 68	230.8 ± 75
Volume of Drainage in the second 24 h (ml)	53.7 ± 3.8	63.3 ± 8.9	63.5 ± 14.2
Total volume of drainage (ml)*	231.5 ± 18.1	248.5 ± 18.8	318.5 ± 26.7

*Data between 3 groups has siginificant difference

The HCT data of each drainage sample are presented in Table 3, from which we learned that on the first day, the average HCT of drainage is 18.04%, which is lower than that in whole blood CBC by a small extent. However, the average drainage HCT reduced over time. On the second day, when the drainage was removed, the average HCT dropped to 11.72%.We also found that the HCT of drainage at different stage were similar for patients in 3 groups.

**Table 3:** The Drainage blood of postoperative drainage

Variable	Group A	Group B	Group C
HCT of Drainage in 24 h (%)	18.04 ± 1.22	20.85 ± 1.16	21.06 ± 1.17
HCT of Drainage in the second 24 h (%)	11.72 ± 1.17	13.22 ± 1.23	12.53 ± 1.24
Drainage blood in 24 h (ml)*	34.95 ± 5.05	39.37 ± 3.79	54.06 ± 5.56
Drainage blood in the second 24 h (ml)	4.85 ± 0.66	7.39 ± 1.07	7.79 ± 3.58
Total volume of drainage blood (ml)*	39.62 ± 5.13	47.53 ± 6.08	61.84 ± 6.60

*Data between 3 groups has siginificant difference

There were no perioperative complications, such as deep venous thrombosis (DVT)/pulmonary embolism (PE), postoperative hematomas/seromas, and postoperative infections, in the 3 groups.

## Discussion

Patients undergoing spinal fusion surgeries are at risk of large amounts of blood losses, which may result in potential risks for subsequent postoperative hemodynamic instability, blood transfusions, and delayed recovery. Rapid and effective hemostasis during the operation allows the surgeon to retain visualization of the surgical sites, thus minimizing the potential injuries to nerve roots and reducing procedure durations. At the same time, effective intraoperative hemostasis plays a major role in reducing morbidity, mortality, and health care costs [4]. Yu-Hua Huang reported that substantial bleeding in lumbar fusion is associated with higher incidences of morbidities and prolonged length of hospital stay [5].

Hemostatic sponges and gelatin sponges are hemostatic material currently offered as prophylactic agents to reduce surgery-associated blood loss. Gelatin sponge is highly absorptive, expansile, and works through mechanical hemostatic mechanism [6]. Jian Wu reported that application of absorbable gelatin sponge at the end of multilevel posterior lumbar fusion can significantly decrease postoperative drain outputs and length of hospital stays [7]. Samuel K. Cho, on that basis, added the known coagulation factor II, thrombin, into gelatin to endow it with both mechanical and chemical hemostatic properties [8]. Their results showed that the thrombin-soaked gelatin sponges would further reduce postoperative bleeding and subsequent drain output [9]. However, the preparation of thrombin-soaked gelatin sponge is complex and time-consuming, which may increase operative durations and anaesthetic risks [10]. The hemostatic sponge has characteristics of both thrombin and gelatin at the same time, so it is widely used in various kinds of surgeries. However, there remains no consensus regarding its efficacy in lumbar spinal surgeries.

In our study, those received hemostatic collagen sponges exhibited lower postoperative drainage than those received gelatin sponges in the first 24 h. However, volume of drainage in the second 24 h was similar between the 3 groups. We inferred that on the second day, the postoperative bleeding is less and the effects of three hemostasis materials were not lastingly significant. Previous studies referred the drainage volume as a main measurable index of postoperative blood loss. However, the transfusion for blood loss after surgery depends not only on the fluid loss amount but also on the pure blood contain in drainage. We believe that postoperative drainage volume does not necessarily equal to postoperative blood loss because the composition of drainage varies from person to person and also with time-lapse after operations [11]. These differences can be reflected through some parameters, such as HCT and HGB of drainage.

According to our investigation, HCT of postoperative drainage in 24 h always fluctuates in a certain range between 15% to 25%, with HGB reaching a maximum of 80 g/L. However, after 24 h, the HCT and HGB of drainage declined apparently, sometimes HGB was less than 10 g/L. These results demonstrated that the blood contained in drainage decreases while other components, such as tissue fluid, becomes the major constituents gradually.

**Fig. 3:**
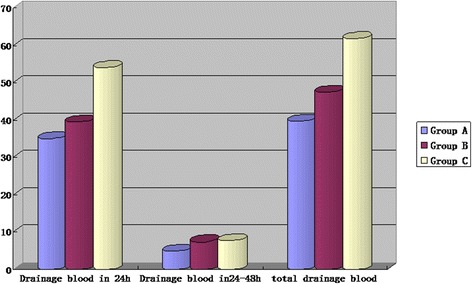
Comparison of postoperative drainage blood

The primary function of hemostatic collagen sponge is bleeding control, therefore, applying a precise parameter to calculate the real volume of blood loss in drainage is necessary. We examined CBC test for drainages and recorded the HCT and HGB of the fluid. Then we calculated the drainage blood to estimate patients’ postoperative blood loss. The calculation formula is: drainage blood = volume of drainage × HCT [12]. We compared postoperative drainage blood of 3 groups in Fig. 3.

As a result, we did not find statistically significant differences of drainage HCT and HGB between the three groups. As for blood in drainage, group A and group B appeared less than group C because of different drainage amount, but no differences were found between group A and group B. These results suggested that the hemostatic collagen sponge can reduce operative bleeding without influencing blood contained in drainage.

## Conclusion

Our study suggests that hemostatic collagen sponge demonstrated better hemostasis effects than gelatin sponge with lower volume of postoperative drainage volume and blood loss in posterior spinal fusion surgery. These findings are important to note as surgeons are under increasing pressure to minimize costs and streamline the entire surgical experience for spinal fusion surgery. We believe that hemostatic collagen sponge is an effective tool in reducing the surgical bleeding and its associated risks. However, careful surgical technique always remains crucial to the success of surgery, which cannot be replaced by sole use of hemostatic material.

## Abbreviations

BMI: Body mass index
CBC: Complete blood count
EBL: Intraoperative estimated blood loss
HCT: Hematocrit
HGB: Hemoglobin
